# Progress in Surgery

**Published:** 1898-06-18

**Authors:** 


### Progress in Surgery.

### SURGERY OF THE CHEST.

(*Concluded from page* 185.)

**Treatment of Pneumo-thorax and Pyo-pneumothorax.**—Dr. Hopkins also detailed two cases of traumatic pneumothorax in which aspiration had been of benefit. The first case, a young man, had a general pneumothorax secondary to a fracture of a rib. A large quantity of air was drawn off. Physical signs then demonstrated the restoration of the lung, and there was no recurrence of the pneumothorax.

The second case was that of a Chinaman who received a stab wound two inches long in the seventh space on the left side. The lung could be seen through the wound lying collapsed posteriorly. There was extreme shock. He was transfused, and strychnine given, and the air removed from the pleura by aspiration through a short piece of rubber tube fixed in an airtight manner by suturing the wound around it and sealing a phlange around with collodion and strapping. The air being removed, the tube was flexed and tied, and in four days removed without air again entering the pleura. The case was very successful.

At the November meeting of the Medical Society of London, Dr. West[4] read a paper on pyo-pneumothorax, in which he advocated treating the cases as empyemata are treated, viz., by drainage. He considered that incision might be required in the early stages of pyo-pneumo-thorax if the dyspnœa and symptoms were urgent, and not relieved permanently by paracentesis; and in the later stages, always if the fluid effusion were purulent, and sometimes when the fluid was serous and very chronic. As a rule these cases are left alone with the idea that the lung will not expand. Dr. West described a case of some months' duration which drainage completely cured, the lung expanding quickly. It is obvious that the success of drainage in these cases will be dependent upon the capability of the lung to expand.

**Pulmonary Abscess.**—Dr. Arnold Edwards[5] records the successful treatment of a case of this kind. The patient a woman of 21, had been ill for about two months with symptoms at first of pneumonia, with latterly abundant fœtid expectoration. She was thin, flushed, short of breath, with a husky and weak voice, a paroxysmal cough, and expectorating a pint of thin, watery, highly offensive pus daily. Temperature, 102 deg., Fahr.; pulse, 128. There were râles all over the chest, but these were larger just below the angle of the right scapula, and the breathing was bronchial there. Exploration in this area yielded a syringeful of pus. An operation was performed the next day. Exploration showed a serous effusion in front on the same side. A portion of the ninth rib was removed where pus had been extracted, and from deep in the lung about 10 ounces of pus were evacuated from a cavity. A tube was left to drain the abscess. The sputum had become almost odourless the next day. Horribly offensive pus had escaped into the dressing. The wound closed in six weeks.

**Hernia of the Lung.**—Mr. Frank Wightman[1] reports an interesting case of this kind. A man, aged 41, was suddenly, while standing in the street, seized with a pain like the "stab of a knife" in the back. The day before he had played the trombone. After three days in bed he began work again, but he still had pain off and on, and about three weeks later discovered a swelling at the seat of the pain. The swelling was $3\frac{1}{2}$ in. by 2 in., opposite the seventh and eighth dorsal spines, and $1\frac{1}{2}$ in. from them on the left side. It felt like a lipoma under the muscles, and was not tender. It had a solid edge, and was lobulated. It was exposed by an incision, and found to lie beneath the trapezius and erector spinæ. No communication with the cavity of the chest existed. No relief of the pain followed its removal. A section showed it to be a portion of collapsed lung. Mr. Wightman remarks upon the unusual position of the hernia, and the peculiar way in which it had become isolated. These hernias are produced during forcible expiration, and may be spontaneous, as in this case, traumatic, or congenital, and are generally in front or at the side of the chest, where the intercostal spaces are wider, and the muscular coverings less substantial.

[4] Lancet, Nov., 1897. [5] Lancet, Dec., 1897.

### CRANIAL SURGERY.

**Complications of Middle Ear Disease.**—The serious results which so frequently follow on neglected inflammations of the middle ear renders this subject one of perennial interest, not only to the surgeon, but even more to the general practitioner, whose lot it usually is first to meet these cases. It is now pretty generally recognised that epidemic influenza furnishes a considerable quota of these conditions, and in the current literature of recent weeks a number of such instances have been recorded.

**Mastoid Disease.**—At the recent meeting of the American Medical Association held at Philadelphia, Milbury[1] read a paper on diseases of the mastoid. In considering the anatomy of this process and its surroundings the author throws doubt on all the speculations regarding the location of internal parts from external measurements, shape and type of skull, &c. A primary periostitis, independent of middle ear disease, occasionally occurs, but is exceedingly rare. When pus is pent up in the mastoid antrum it may find vent in one of four directions—through the outer mastoid wall or into the external meatus, through the digastric fossa, into the middle fossa of the base of the skull, or into the groove for the lateral sinus. When the pus escapes through the digastric fossa an abscess forms under the sterno-mastoid muscle, and may pass deeply down the neck. An example of this is reported by Ouston[2] in a girl aged 15, who had suffered from ear discharge for twelve years. For six months pain in the head continued, and a swelling in the neck developed. When seen, this swelling showed signs of acute inflammation, and extended from the vertex in the parietal region to the level of the thyroid cartilage, in both the anterior and posterior triangles. On evacuating this purulent collection a considerable area of the mastoid was found necrosed, and the lateral sinus was exposed and accidentally opened. It was not thrombosed. An extra dural abscess also existed, probably, the author thinks, associated with cerebellar mischief. Convalescence was delayed by a degree of basal meningitis, which, however, cleared up, and recovery took place.

In the treatment of mastoid inflammation, Milbury places considerable value on the use of Leiter's coil continuously applied for a period up to forty or fifty hours. He has not found, however, that in influenzal cases this measure arrests the formation of pus. If not efficacious in fifty hours, operation should be resorted to. In performing such operations as Schwartze's or Stacke's, the author emphasises the importance of persevering till sound bone is found in every direction, even at the risk of exposing the dura mater, a contingency which he does not look upon as serious. He believes in the value of syringing in these cases, contrary to the teaching of Küster and Bergmann. R. W. de Santi[3] communicates a case, interesting in several points. The patient, a woman aged 21, suffered from more or less persistent purulent ear discharge for fourteen years, in spite of vigorous and thorough skilled treatment. In the end, symptoms indicative of intracranial mischief supervened, and De Santi explored the brain with absolutely negative results as regards the finding of any pathological condition. The symptoms, however, cleared up, and the patient was much improved for ten months, when the mischief recurred. On making a second exploration, a very chronic abscess was found between the wall of the lateral sinus and the petrous bone. The sinus was not thrombosed.

Downie,[4] of Glasgow, records a post-influenzal case of extensive septic thrombosis of the lateral sinus occurring in a man of sixty-six. The primary influenza dated five years back, since when the patient had been deaf in the right ear with, however, little or no discharge for some months. A mastoid periostitis supervened, and although this was incised intracranial symptoms developed. On exposing the mastoid region a piece of necrosed bone was found overlying the lateral sinus, on the removal of which pus escaped from that channel. The jugular vein was not ligatured. Recovery was rapid and complete.

Dahlgren[5] reports three cases where operation was successful in thrombosis of the lateral sinus. In all of them the internal jugular vein was ligatured. In one acute purulent inflammation of the middle ear following influenza was the cause; in another blocking of the middle ear by a polypus produced a chronic discharge and mastoiditis; in the third a simple acute otitis media started the process.

[1] Med. Rec., Nov. 13, 1897. [2] British Med. Jour., Jan. 22, 1898. [3] Lancet, Jan. 29, 1898. [4] Lancet, Jan. 1, 1898. [5] Arch. für Klin. Chir. Bd. iii. heft 3.

---

### SURGERY OF THE PERITONEUM.

**The Management of Patients** before and after laparotomy is discussed by Wiggin,[1] who maintains the importance of preparatory treatment, and concludes: (1) The administration of cathartics, followed by large enemata, is important as a preparatory measure. (2) The urine and temperature should be examined in all cases before operation. (3) In the female, operation is best performed a few days after a menstrual period, and the vagina should be cleansed even if the abdominal route only is to be employed. (4) One ounce of peptonised food containing stimulants should be administered two hours before the anæsthetic to check nausea and vomiting after operation. (5) During operation the patient's body should be properly protected with clothing and blankets. (6) The pulse should be stimulated before the heart has become much exhausted, and intravenous saline injections given before the radial pulse is extinct. (7) A quantity of hot saline solution left in the abdomen stimulates the patient, prevents adhesions, and lessens the danger of septic infection of the peritoneum. (8) Food should be administered early in reasonable quantities. (9) Stimulating enemata should be withheld after operations, in which extensive and firm pelvic adhesions have been broken up. (10) As soon as the diagnosis of intestinal paresis is made, the stomach should be washed out and saline cathartics persisted in till the bowels move. (11) Cathartics should not be administered too early in those cases pursuing a normal course. Dudley[2] believes the Trendelenburg posture to be unnecessary and dangerous owing to the extra pressure put upon the heart and lungs and on the cerebral circulation.

**The Abdominal Incision** is considered by Woolsey,[3] who concludes: (1) That abdominal incisions, except those in or close to the median line, should be obliquely transverse in order to parallel the nerves (and thereby also the cleavage lines of the skin), so as to avoid partial paralysis of the muscles, weakness of the abdominal wall, and a tendency to hernia. (2) That intermuscular, or even transmuscular incisions, should be preferred to those in the linea alba or semilunaris, for in both the latter cases the cicatrix is less strong and more prone to hernia, and in the semilunar line the nerves are necessarily divided. 3. That in place of the median vertical incision, the inter-muscular incision near the inner margin of the rectus, or the trap-door incision around this inner margin offers many important advantages. He quotes Ramsay (*v. Lancet*, November 30th, 1895) to show that the slight vascularity of the linea alba is really a disadvantage, because it tends to delay rapid and firm healing, and therefore predisposes to hernia. Volkovitch[4] also recommends an incision through either rectus in place of the incision along the linea alba. The wound is closed by a series of sutures at different depths. The first series of interrupted sutures takes in the peritoneum and the inner layer of the rectal sheath. The second series unites the remaining layers with the exception of the skin, which is stitched up separately by a continuous suture. In operations upon the lateral regions of the abdomen, the principle to be observed is to separate the tendino-muscular structures along the course of their fibres, as each layer comes into view, and not to divide the muscular fibres across in the plane of the wound as is usually done. Lennander[5] describes a new method of incision, which consists of a vertical section of the anterior layer of the sheath of the rectus and free exposure without incision of this muscle, the outer margin of which is pushed inwards in the lateral operation, and the inner margin outwards in a median laparotomy. The posterior layer of the sheath thus exposed is incised in the same direction, and to the same extent, as the wound in the skin. In closing the abdominal wound four rows of sutures are applied, one to the peritoneum and posterior layer of the sheath, the second for fixing the replaced margin of the rectus to the corresponding margin of the sheath, the third for closing the wound in the anterior layer of the sheath, and the last for bringing together the edges of the skin.

**Hernia of the Abdominal Cicatrix** is best treated, according to Doran,[6] in practically all cases by opening the peritoneum, and thus he opposes the views advocated by Greig Smith. Tense narrow necks may form in these cases, and secondary sacs and burrowing of omentum and intestine are observed in this form of hernia just as in umbilical hernia. Firm adhesion of intestine to the cicatrix is frequent. For these reasons he prefers to open the sac. The best method of suturing the abdominal wound after operation cannot be said to be settled, but the author always applies deep interrupted sutures and takes up not more than one-eighth of an inch of the peritoneum.

**Contusions of the Abdomen**, with serious symptoms can, according to Michaux,[7] only be diagnosed and treated by laparotomy, the principal rules for which are: (1) Laparotomy as early as possible after shock has passed; (2) rapid operating, evacuation of blood with the aid of large sponges, methodical exploration of the gut, evisceration only when one finds alimentary *débris* or fæcal matter; (3) leave the abdomen open to a large extent at the end of the operation, protecting dangerous areas with iodoform gauze.

**Peritonitis** of the chronic form, and especially of the traumatic or post-operative types, is best treated, says Largeau,[8] by abdominal section. Care must be taken that the disease is not assuming a temporarily acute form when the abdomen is opened. Mere exploration has given good results after all forms, but Largeau admits that where there is ulceration and suppuration the prognosis is bad. In the ascitic and dry forms of tuberculous peritonitis abdominal section is alike satisfactory. The lower end of the incision should be brought low down in a female subject, as tubercular appendages, which are often the seat of the primary mischief, may require removal. The main part of the operation is thorough drying of the exposed cavity with fine sponges or compresses. Not only Douglas's pouch must be so treated, but care must be taken to dry all diseased peritoneum lying under coils of intestine. The primary cause must be sought, the gravest condition being an ulcerated area beginning in the small or large intestine. When this condition is detected pus is generally present around the breach in the intestinal wall. This pus must be washed away; it is useless to attempt to close the intestine by suture, hence drainage is compulsory. Lastly, in the purely dry form of tuberculosis peritonitis separation of adhesions often proves satisfactory, as they are firm in this type, and give rise to obstruction. Washing out with chemical solutions, or flushing of the peritoneum, seems superflous, and drainage is only needed when the intestine is ulcerated.

1 Med. Rec., Jan. 22, 1893, p. 115. 2 Ibid., p. 132. 3 Annals of Surgery. Jan. 1898. 4 Brit. Med. Jour. Epit, March 5, 1898; and Vratch, No. 5, 1898. 5 Brit. Med. Jour. Epit., Feb. 5, 1898; and Centralbl. f. Chir., No. 4, 189[illegible]. 6 Lancet, Nov. 27, 1897, p. 1,379. 7 Annals of Surgery, March, 1898, p. 390. 8 Brit. Med. Jour. Epit., Feb. 5, 1898.

